# Supplementary material for: T-Cell Defects Associated to Lack of Spike-Specific Antibodies after BNT162b2 Full Immunization Followed by a Booster Dose in Patients with Common Variable Immune Deficiencies
Source: Cells. 2022 Jun 14;11(12):1918. doi: 10.3390/cells11121918 (PMC9221747; doi:10.3390/cells11121918)
Supplement: Supplementary file 1 [file cells-11-01918-s001.zip › cells-1742439-supplementary.pdf]

Table S1. SARS-CoV-2 infection in CVID patients immunized with three doses of BNT162b2 vaccine

Group	ID	days from booster dose	COVID-19 treatment	Days of qRT PCR positivity	Severity
NR	1	88	Bamlanivimab/etesevimab	24	mild
	3	157	Sotrovimab	8	mild
	4	176	Sotrovimab	45	mild
	6	95	Sotrovimab	41	mild
	10	117	Sotrovimab	37	asymptomatic
	14	195	Paxlovid	14	mild
	15	116	Sotrovimab	25	mild
	17	150	Paxlovid	7	mild
S1 IgG-R	20	121	Sotrovimab	26	mild
	26	87	No treatment	8	asymptomatic
	27	88	Sotrovimab	26	mild
	34	183	Sotrovimab	9	mild
S1 IgG/IgA-R	37	101	Sotrovimab	14	mild
	38	130	Sotrovimab	11	mild
	42	117	Sotrovimab	16	mild
